# Supplementary material for: Cognitive Impulsivity in Anorexia Nervosa in Correlation with Eating and Obsessive Symptoms: A Comparison with Healthy Controls
Source: Nutrients. 2024 Apr 13;16(8):1156. doi: 10.3390/nu16081156 (PMC11054498; doi:10.3390/nu16081156)
Supplement: Supplementary file 1 [file nutrients-16-01156-s001.zip › nutrients-2866009-supplementary.pdf]

Supplementary material S1

Table S1. Pearson's correlations of *Total* and *Cognitive Impulsivity* in healthy controls (N=59)

	**BIS-11 Global Score**		**BIS-11 Cognitive Instability**	
	Pearson's coefficient	p	Pearson's coefficient	p
Age	-.039	.770	- .102	.442
BMI	-.040	.762	.137	.301
OCI Washing	.100	.451	**.267**	**.041**
OCI Checking	.022	.867	.256	.050
OCI Doubting	.078	.555	**.367**	**.004**
OCI Ordering	.083	.531	.161	.222
OCI Obsessing	.022	.860	**.440**	**< .001***
OCI Hoarding	.126	.342	**.293**	**.025**
OCI Mental Neutralizing	.043	.747	.272	.037
OCI Global Score	.091	.494	**.344**	**.008**
STAI State Anxiety	.084	.579	-.032	.835
STAI Trait Anxiety	.160	.289	.067	.659
BDI	.017	.907	.113	.439

Legend: BIS-11 = Barratt Impulsiveness Scale version 11; BMI = Body Mass Index; OCI = Obsessive Compulsive Inventory; STAI = State-Trait Anxiety Inventory; EDI–2 = Eating Disorders Inventory—2; BDI = Beck Depression Inventory. *=significant after Bonferroni-Holm correction.

Table S2. Linear regression model for Obsessive thoughts and behaviors (OCI Total score) in HC individuals (N=59).

	**Multivariate Regression**			**Properties of the Model**		
Variables	Beta	t	p	R	F	p
BIS-11 Cognitive Instability	**0.599**	**3.603**	**.001**			
STAI Trait Anxiety	**0.945**	**2.208**	**.034**			
EDI–2 Bulimia	**3.938**	**2.204**	**.034**			

EDI–2 Drive for Thinness	-.1.743	-1.223	.229			
BDI	-0.364	-0.477	.636			
STAI State Anxiety	0.169	0.434	.667	**.642**	**6.978**	**.001**
Age	0.459	0.322	.749			
BMI	-0.574	-0.296	.769			
EDI–2 Body Dissatisfaction	-0.106	-0.287	.853			

Legend: HC = Healthy Controls; BIS-11 = Barratt Impulsiveness Scale version 11; BMI = Body Mass Index; OCI = Obsessive Compulsive Inventory; STAI = State-Trait Anxiety Inventory; EDI–2 = Eating Disorders Inventory—2; BDI = Beck Depression Inventory.
